# Ferromagnetic Behavior and Magneto-Optical Properties of Semiconducting Co-Doped ZnO

**DOI:** 10.3390/nano12091525

**Published:** 2022-05-01

**Authors:** Antonio Di Trolio, Alberto M. Testa, Aldo Amore Bonapasta

**Affiliations:** 1 CNR-Istituto di Struttura della Materia, Via del Fosso del Cavaliere 100, 00133 Roma, Italy; 2 CNR-Istituto di Struttura della Materia, Via Salaria Km. 29,300, 00015 Monterotondo, Italy; albertomaria.testa@mlib.ism.cnr.it (A.M.T.); aldo.amore@mlib.ism.cnr.it (A.A.B.)

**Keywords:** ferromagnetism, Co-doped ZnO thin films, hydrogen incorporation, transparent conducting oxides

## Abstract

ZnO is a well-known semiconducting material showing a wide bandgap and an *n*-type intrinsic behavior of high interest in applications such as transparent electronics, piezoelectricity, optoelectronics, and photovoltaics. This semiconductor becomes even more attractive when doped with a few atomic percent of a transition metal. Indeed, e.g., the introduction of substitutional Co atoms in ZnO (ZCO) induces the appearance of room temperature ferromagnetism (RT-FM) and magneto-optical effects, making this material one of the most important representatives of so-called dilute magnetic semiconductors (DMSs). In the present review, we discuss the magnetic and magneto-optical properties of Co-doped ZnO thin films by considering also the significant improvements in the properties induced by post-growth irradiation with atomic hydrogen. We also show how all of these properties can be accounted for by a theoretical model based on the formation of Co-V_O_ (oxygen vacancy) complexes and the concurrent presence of shallow donor defects, thus giving a sound support to this model to explain the RT-FM in ZCO DMSs.

## 1. Introduction

Zinc oxide (ZnO) exhibits remarkable properties such as a large band gap (3.4 eV), long spin coherence length (10.7 nm at 10 K), and large exciton binding energy (60 meV), which are very interesting qualities in the fields of spintronics, transparent electronics, piezoelectricity, and optoelectronics [1,2,3]. Moreover, the discovery of room-temperature ferromagnetism (RT-FM) in transition metal-doped ZnO [4,5,6,7,8,9,10] has permitted the inclusion of this material in the DMS (diluted magnetic semiconductor) family [11], thus broadening the possibility of its application in modern technology. In particular, over the past few decades and still recently, a number of theoretical and experimental studies have investigated the effects of the introduction of Co atoms in substitution for the Zn ones (Co_Zn_) on the magnetic properties of ZnO [12,13,14,15,16,17,18,19,20,21,22,23,24,25,26,27,28,29,30,31,32,33,34,35,36,37,38,39,40,41,42,43,44,45,46,47,48,49,50,51,52,53,54,55,56]. However, despite numerous research efforts, the origin of ferromagnetism in Co-doped ZnO (ZCO) is still a mystery, thus acting as a serious obstacle to progress in its applications. Most previous studies have attempted to explain the ferromagnetism and magnetic phenomena observed in ZCO in terms of magnetic clusters [57,58,59,60,61,62,63], carrier mediation [64,65,66], or magnetic polarons based on intrinsic defects [67,68,69,70]. None of these studies could exhaustively explain the reported results. Remarkably, some years ago, an experimental and theoretical study was published which gave support to a model based on the formation of Co-H-Co complexes. This model seems to explain RT-FM in heavily Co-doped (more than 10% Co) ZnO [69]. However, such a model does not account for the RT-FM observed in ZCO doped with the smaller percent of Co typical of DMSs [8,10,16,20,21,24,25,45,71].

In this context, we have chosen to propose a somewhat “unconventional” review, with the principal aim of carefully discussing a collection of previous results spanning from magnetic to transport and surface ZCO properties, and to show how all of them can be accounted for by a *unifying* theoretical model, thus giving a sound support to this model to explain the RT-FM in ZCO DMSs. Our model is based on the formation of Co-V_O_ complexes in the presence of shallow donor defects. It works in lightly doped ZCO, thus providing, together with the Co-H-Co model, a worthy, complementary explanation of the RT-FM in ZCO. More specifically, we consider the profound effects that Co doping has on the magnetic, magneto-optical (MO) [24,72,73,74,75], and transport properties of polycrystalline ZnO [76], as well as on its surface reactivity [77]. These effects will be discussed by considering the role that native defects play in their occurrence, such as oxygen vacancies (V_O_) and Zn interstitials (Zn_int_). Moreover, attention will be devoted to the significant improvements in the ZnO properties that occur when Co doping is combined with a post-growth irradiation of the ZCO samples with atomic hydrogen, HZCO [25]. 

A variety of techniques have been exploited for DMS growth, such as pulsed laser deposition (PLD) [12,13,14,15,16,17,73], sol gel [18], MOCVD [19], chemical routes [20,21], and sputtering [22,23]. We will concentrate on the properties of polycrystalline ZnO (ZCO) thin films grown by PLD. In the following sections, the growth and hydrogenation methods of polycrystalline ZCO films used in our studies will be introduced, together with a brief description of the experimental techniques employed for their morphological, structural, and magnetic characterization. Then, experimental results regarding the magnetic and magneto-optical properties, as well as other properties of the investigated ZCO and HZCO films, will be reported and discussed in light of the proposed theoretical model. 

## 2. Growth, Structural and Magnetic/MO Characterization of ZCO Thin Films

### 2.1. ZCO Growth and Microstructure 

In PLD, the growth of polycrystalline thin films takes place from the formation of a gaseous plasma. A pulsed and focused high-power laser beam is used to ablate material from a target having the required stoichiometry. The laser–matter interaction generates a plasma plume containing atoms, ions and molecules, which expand in vacuum with kinetic energies of ~100 eV towards a substrate where nucleation and film growth take place. The stoichiometry transfer between the target and the film, as well as the possibility of working in the presence of inert and reactive gas atmospheres up to pressures in the mbar range, make PLD a very attractive technique.

In the following, we report a summary of PLD growth as well as the structural and microstructural characterizations of ZCO thin films, extensively discussed elsewhere [24,75]. Co-doped ZnO thin films were epitaxially grown on Al_2_O_3_ (001) substrate by PLD using a Nd:Yag laser, operating at 355 nm, with a fluence of 2 J/cm^2^. Targets were prepared from analytically pure Co_2_O_3_ and ZnO powders, which were mixed with nominal compositions of 4 at.%, 5 at.%, and 6 at.%. The mixtures were calcined with solid state routes at 400 °C for 10 h in air, pressed into pellets, and then sintered at 500 °C for 20 h in air. During the deposition, O_2_ was fluxed to reach the desired background pressure in the 10^−5^ mbar range. The substrate temperature was set at 500 °C, a limiting value to avoid the Co metal clustering. Film thickness, measured by an α-step profilometer, ranged from 150 to 600 nm. Hydrogen incorporation was obtained by post-growth-irradiating ZCO films with a beam of hydrogen ions of 100 eV, produced by a Kaufman source using current densities of the order of tens of A/cm^2^. An H^+^ dose of 4.5 × 10^18^ ions/cm^2^ was used to irradiate the film at T = 400 °C. [25,73]. 

The film structure was investigated by means of the X-ray diffraction (XRD) measurements of Bragg–Brentano geometry, using Cu Kα radiation. The films mainly showed a (001) preferential orientation, with the *c*-axis of the hexagonal lattice perpendicular to the Al_2_O_3_ substrate [73,74]. Combined XRD, scanning electron microscope (SEM), and transmission electron spectroscopy (TEM) analyses revealed a columnar growth of the ZnO grains through the entire film. The XRD spectra [74] showed an almost singular presence of the ZnO (002) peak, whereas the *c* lattice parameter, estimated from the analysis of the angular position of the ZnO (002) peak, was quite close (within 0.2–0.4%) to the ZnO compound nominal value (ICDD file (no. 36–1451)). Moreover, the average lateral grain size was estimated between 40 and 100 nm by using the full width at half maximum (FWHM) of the ZnO (002) peak in the Scherrer formula. TEM analysis with selected area electron diffraction measurements showed an oriented growth of ZCO on Al_2_O_3_ substrates with the orientation relationships: [010]ZnO//[–110] Al_2_O_3_, [−110]ZnO//[−110] Al_2_O_3_, and (001)ZnO//(001) Al_2_O_3_.

### 2.2. Magnetic and MO Properties

Volume magnetization vs. field measurements were performed at 300 K using a Micro Sense Model-10 VSM magnetometer with a maximum field of 20 kOe. Room temperature optical transmittance spectra were acquired using a Jasco V670 spectrophotometer. A home-built apparatus was used to investigate MO properties in the wavelength range 350–850 nm both in the Faraday rotation and magnetic circular dichroism configurations under magnetic fields up to 1.3 T [74]. Local magnetization measurements were performed at 300 K by a MO Kerr magnetometer at wavelengths of 660 nm in longitudinal geometry (as the investigated films exhibited an in-plane magnetic anisotropy only) with a max field of 0.46 T, enabling us to image magnetic domains to investigate the magnetic (coercivity, squareness ratio) and MO (Kerr rotation angle, reflectivity) properties. The scanning laser microscope (SLM) technique was used for imaging: in this approach, a 660 nm semiconductor laser was detected by a computer-controlled couple of galvanometric mirrors, allowing us to select different points on the sample surface and to analyze local magnetic properties. In particular, by selecting arrays of 50 μm × 200 μm (with a 5 μm step) on the surface, a spatial mapping of the sample properties was obtained. In particular, the mapping procedure consisted of measuring the characteristic quantities of a large number of Kerr hysteresis loops for each selected point. In this way, two-dimensional spatial distribution (color) maps of local sample properties could be generated from every local area (cell), simultaneously measured with identical field sweeping (frequency of 2.1 Hz) conditions to guarantee the largest reproducibility of the measurements. 

## 3. Results 

### 3.1. Role of Defects and n-Carriers in the ZCO Ferromagnetic Behavior

**Figure 1:**
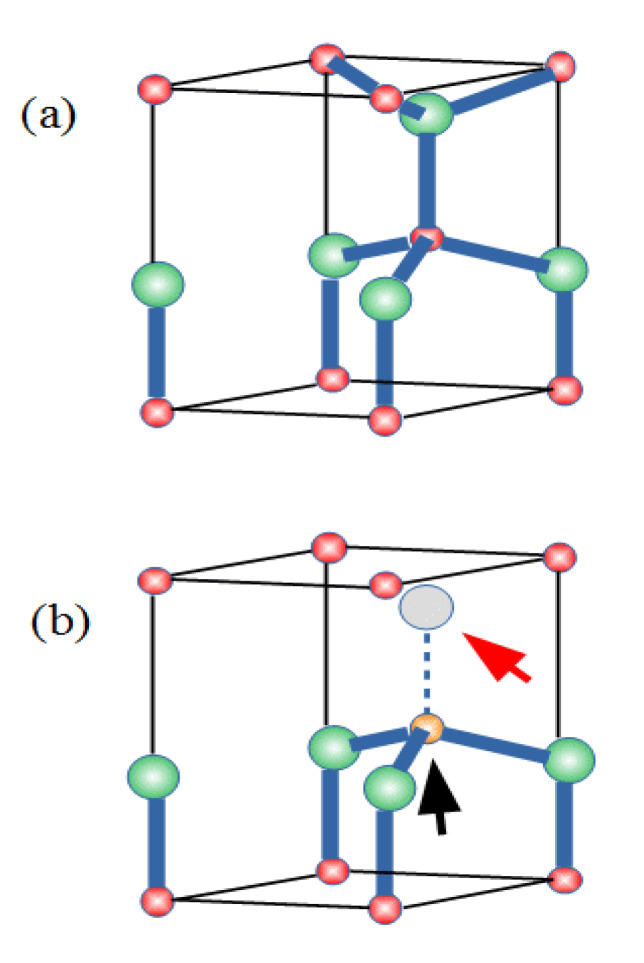
(**a**) ZnO Wurtzite structure. Red and green spheres represent Zn and O atoms, respectively. (**b**) The black and red arrows indicate the positions of a Co atom (orange sphere) substituting a Zn one and a missing O atom (grey sphere) in a Co-V_O_ complex, respectively.

The polycrystalline films investigated here were formed by ZnO grains, showing the wurtzite structure of the ZnO crystal (Figure 1a). One Zn atom was surrounded by four O atoms at the vertices of an almost regular tetrahedron. Spectroscopy confirmed that a Co^2+^ substituting Zn^2+^ is the center responsible for RT-FM in ZCO [77]. Moreover, the oxygen-deficient growth of ZCO promotes RT-FM [8,14,34]. Similarly, annealing in a vacuum enhances the magnetic moment and produces ferromagnetism [7,27,28], while annealing in oxygen has the opposite effect [19,29,30].

**Figure 2:**
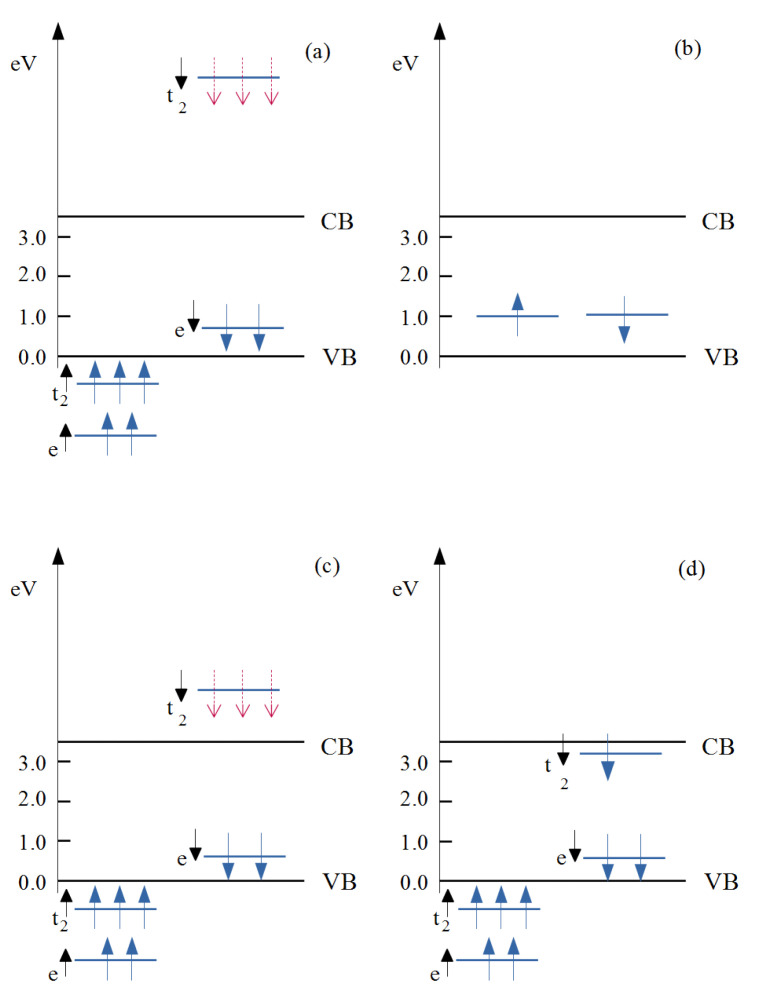
Schematic view of the electronic states induced in the ZnO band gap by: (**a**) a Co atom substituting a Zn one, (**b**) an oxygen vacancy, V_O_, (**c**) a Co-V_O_ complex, and (**d**) a negatively charged Co-V_O_^−1^ complex. Red–dashed arrows indicate unoccupied, minority spin states.

These results suggest a role of the intrinsic defects favored by these experimental conditions, such V_O_ and Zn_int_, in the occurrence of RT-FM. V_O_s and Zn_int_s are predicted to behave as deep and shallow donors, respectively [31]. Zn_int_s could account for the native *n*-type behavior generally shown by ZnO, although their formation has been somewhat questioned [31]. Regarding the oxygen vacancies, the role of Co-V_O_ complexes in the appearance of RT-FM was firstly proposed in a theoretical study [32]. The results of that study, further refined in a subsequent paper [78], indicated electronic structures for an isolated, substitutional Co^2+^, an isolated V_O_, and a Co-V_O_ complex, as schematically described in Figure 2. Co^2+^ ions in the Zn site are nominally in a *d*^7^ valence configuration, and the approximately tetrahedral crystal field surrounding the Co atom (see Figure 1a) splits its 3*d* states into a set of lower *e* and higher *t*_2_ levels. The majority-spin *e*^↑^ and *t*_2_^↑^ as well as the minority-spin *e*^↓^ states are filled, while the minority spin (*t*_2_^↓^) was empty (Figure 2a), leading to a net magnetic moment of 3 μ_B_ per Co_Zn_.

**Figure 3:**
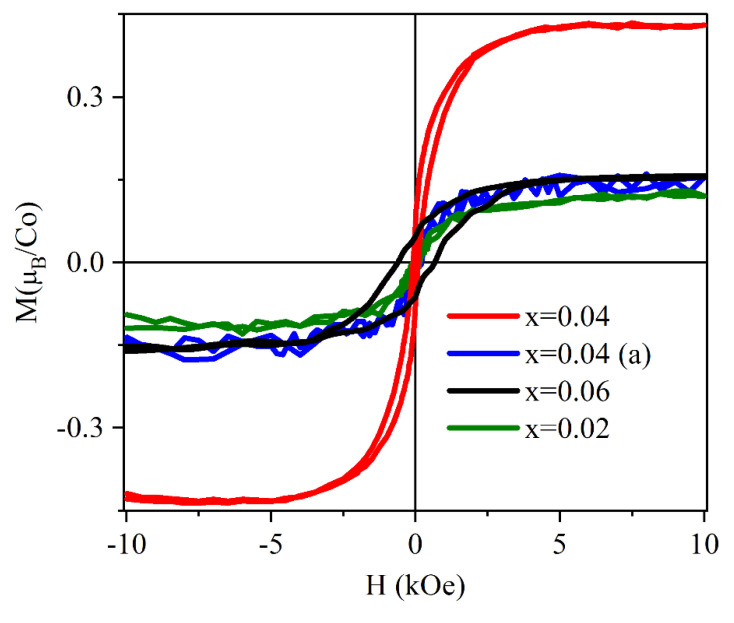
Hysteresis cycle for the four investigated epilayers (after subtraction of the substrate signal); 0.04 (a) indicates an oxygen annealing. Reproduced with permission from [73]. Copyright 2001 American Physical Society.

The energy position of the *t*_2_^↓^ states relative to the host conduction band minimum (CBM) is crucial for the ferromagnetism, since it determines the efficacy of a carrier-mediated mechanism. In fact, generally, the stabilization of the ferromagnetic interaction is related to the electron hopping between transition metal dopant sites hosting partially occupied *d* states. Such a mechanism is active if the *d* states are resonant within either the conduction or the valence band continuum of the host semiconductor. In this situation, their occupancy can be continuously varied. Accordingly, theoretical works in the literature [32] indicate that a partial occupancy of the *t*_2_^↓^ states under electron doping is a minimum requirement for long-range FM interactions between Co ions in ZCO. Such a requirement is not satisfied in the case of Co_Zn_, the *t*_2_^↓^ states being much high relative to the CBM (see Figure 2a). The same theoretical results show a dramatic lowering in the energy of the same *t*_2_^↓^ states when the Co atoms form a Co-V_O_ complex (see Figure 1b for a sketch of the complex structure) from 3.3 eV to 1.3 eV, relative to the CBM (see Figure 2c) [79]. However, the *t*_2_^↓^ states were still too high in energy and the Co-V_O_ complex was no longer considered as a possible cause of FM. On the contrary, a possible role of Co-V_O_ complexes in the appearance of RT-FM was investigated in a further study where Co K-edge XANES (X-ray absorption near-edge structure) and RT magnetization versus field (H) measurements were performed together with theoretical calculations [73]. Three Zn_1-x_Co_x_O epilayers were grown where x = 0.02, 0.04, and 0.06, respectively. A piece of the x = 0.04 sample, termed x = 0.04 (a), was subsequently annealed in oxygen. A clear ferromagnetic behavior was shown by all the samples in Figure 3, with the largest value of the saturation magnetization (about 0.44 μ_B_/Co ) found for the sample with x = 0.04, whereas a decrease was observed for x = 0.06. In the latter case, the larger Co content may favor a random distribution of antiferromagnetically coupled nearest neighboring Co ions, as reported in [8]. 

**Figure 4:**
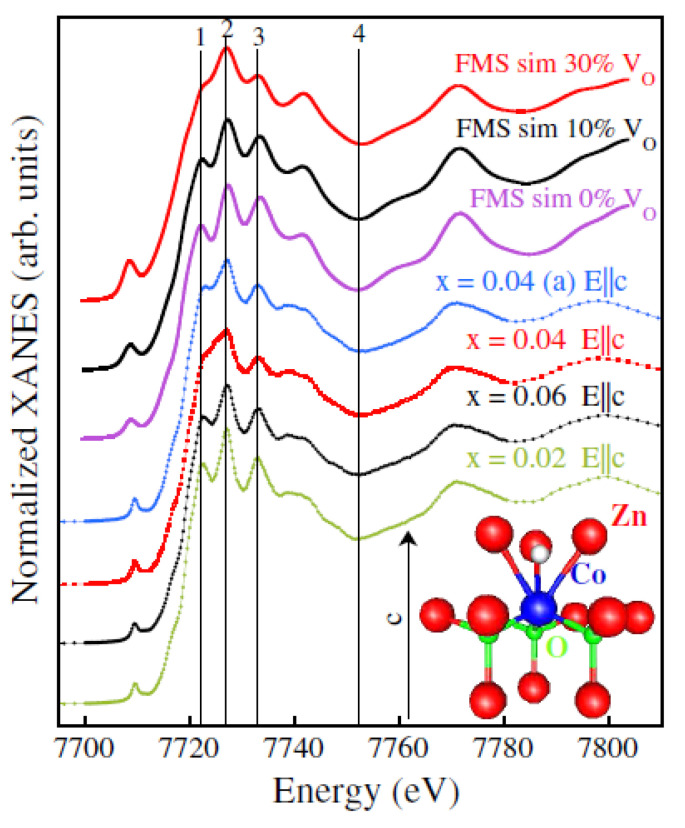
Co K-edge XANES measured with *E*‖ *c* on all samples (bottom spectra) and FMS simulations (top spectra). Inset: Sketch of the Co-V_O_‖ *c* complex used in the simulations. The V_O_ is represented by the white sphere (see also Figure 1b). Reproduced with permission from [73]. Copyright 2001 American Physical Society.

Figure 4 shows the Co *K*-edge XANES spectra of the same samples together with their FMS (full multiple scattering) simulations achieved by calculating the structures of Co_Zn_ and of different, possible configurations of a Co-V_O_ complex by using first principle DFT (Density Functional Theory) methods and assuming in the simulations the substitution of isolated Co with different percentages of the complex. Among the different, investigated defect structures, only that of the Co-V_O_ complex shown in Figure 4 led to a good agreement between the simulated and measured spectra. Then, in the same study, it was considered that: (i) remarkably, the x = 0.04 sample showed the highest values in saturation magnetization; (ii) the valley between features 1 and 2 in the XANES spectrum of the untreated x = 0.04 sample disappeared compared to the x = 0.02 and 0.06 ones; and (iii) the same valley disappeared only in the spectrum simulated with the highest percentage of the Co-V_O_ complex. Already these results clearly indicate that the sample with the strongest ferromagnetic behavior had the highest concentration of Co-V_O_ complexes. As further evidence of the existence of such a relationship, annealing with the oxygen of the x = 0.04 sample induced the recovery of the 1–2 valley, that is, a reduction in the Co-V_O_ complexes and a weakening of ferromagnetic behavior (Figure 3). Thus, that study gave a sound indication that the *presence of* Co-V_O_ *complexes is directly related to the appearance of RT-FM* in ZCO.

**Figure 5:**
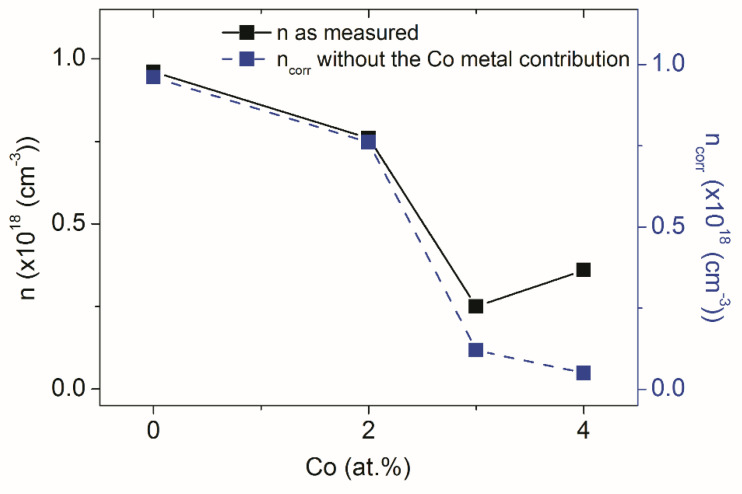
Values of the carrier concentration (n- as measured; n_corr_ - without the Co metal contribution) as a function of Co concentration (percent of Zn atoms) for the C0, C2, C3, and C4 films. Reproduced with permission from [24]. Copyright 2015 Royal Society of Chemistry.

The role of Co-V_O_ complexes in the ZCO magnetic behavior was clarified by a subsequent study focused on the relationships between the conductive and magnetic properties of Co-doped ZnO [24]. In that study, Hall effect and resistivity measurements were performed in the bulk of our Zn_1-x_Co_x_O films, i.e., C0, C2, C3, and C4, with nominal x contents of 0.00, 0.02, 0.03, and 0.04, respectively (Figure 5, Table 1). Table 1 reports values of the investigated electrical and magnetic parameters, whereas Figure 5 shows the carrier concentration (*n*) versus Co concentration (percent of Zn atoms) for the C0, C2, C3, and C4 films. Note that the most significant results in the figure regard the values of the carrier concentrations without the Co metal contribution (*n_corr_*). These experimental measurements provided two unexpected results: (i) a decrease in the electrical conductivity induced by the Co doping; and (ii) the simultaneous occurrence of a reduction in conductivity and an enhancement of ferromagnetic behavior on increasing the Co concentration. The former is a somewhat puzzling result because Co_Zn_, as an isoelectronic impurity with Zn, just adds a fully occupied *e*^↓^ state in the energy gap (Figure 2a), possibly corresponding to a deep donor behavior. Moreover, among the potential defects forming in ZCO, oxygen vacancies behave as deep donors (Figure 2b), Zn_int_ are shallow donors, and only Zn vacancies, V_Zn_, induce acceptor levels that could account for the observed decrease in the *n* carriers. 

**Figure 6:**
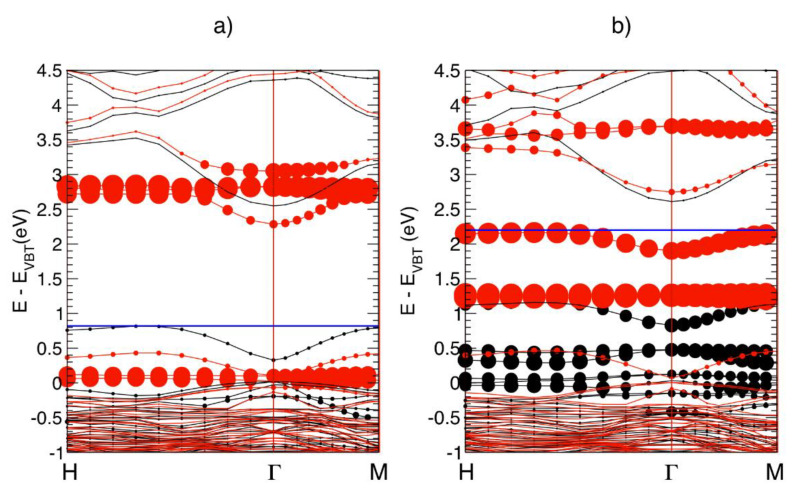
Spin-polarized electronic band structure along the H-Γ-M direction, for the neutral (**a**) and -1-charged (**b**) Co-V_O_ complex in ZnO. Black and red lines correspond the to spin-up and spin-down states, respectively. Circles indicate Co-d contribution to the electronic levels, as circle radii are proportional to the integral of the corresponding charge densities projected on atom-centered orbitals. The horizontal blue line sets the position of the last occupied eigenvalue (Fermi energy). In (**a**), empty levels induced by neutral Co–V_O_ are resonant in the CB, about 0.5 eV higher than the CBM. The zero of the energy scale is the valence band top (E_VBT_). Reproduced with permission from [24]. Copyright 2015 Royal Society of Chemistry.

However, the introduction of Co_Zn_ atoms, close in size to the Zn ones, is expected to induce the formation of Zn_int_ rather than V_Zn_. Regarding the observed decrease in conductivity in correspondence to an enhancement in ferromagnetic order, this result is also quite surprising because it disagrees with the positive correlations between an increase in *n* carrier concentrations and an FM increase reported for Co-doped ZnO films additionally *n*-doped by Zn [19,20,80] or Al [33,34] ions. In order to explain all of these experimental results, theoretical DFT calculations investigated the properties of a charged Co-V_O_^−1^ complex, that is, a complex where the *t*_2_^↓^ state of Figure 2c is occupied by one free electron provided by a shallow donor (recall that shallow donor defects are already present in native *n*-type ZnO). The results of the theoretical simulations are shown in Figure 6 and schematized in Figure 2d.

Occupied *t*_2_^↓^ states descend in energy, moving close to and below the CBM, as independently confirmed by transition state estimates [24]. Thus, the Co-V_O_ empty states can subtract electrons from the CBM by giving rise, at the same time, to an occupied *acceptor impurity band* (AIB) partially mixed with the CB states. Remarkably, this band satisfies the requirement to produce the long-range magnetic coupling of Co dopants discussed above. On such a basis, a well-posed theoretical model can be set up to fully account for the *decrease* in conductivity coexisting with the *enhancement* in ferromagnetic order. 

The AIB model is basically founded on two ingredients: the presence of a defect complex involving an *under-coordinated* Co atom (e.g., surrounded by three O instead of four O atoms, as in the Co-V_O_ complex, see Figure 1b and Figure 4); and the availability of shallow donors providing free electrons. The Co under-coordination weakens the effect of the crystal field surrounding the Co atom, thus favoring a descent in energy of the empty *t*_2_^↓^ states. These states further descend when occupied with free electrons provided by shallow donors and become mixed with the CBM.

It is useful now to briefly recall the main features of the above-mentioned Co-H-Co model for a comparison with the AIB model. As detailed in [69], the former model requires the formation of Co-Co dimers in the ZnO matrix and the presence or the intentional introduction of atomic hydrogen. The formation of Co-Co dimers is favored by the high Co concentrations considered in that study, whereas it is a rare event in ZCO DMS. Moreover, the formation of such dimers is an obstacle to the main requirement of the AIB model, that is, the realization of under-coordinated Co atoms through the formation of Co-V_O_ complexes. The Co-H-Co model is therefore basically antithetic to the AIB one, and it is not surprising that the study in [69] reports an irrelevant role of oxygen vacancies in the FM of highly doped ZCO. In conclusion, the two models fill knowledge gaps on the origin of RT-FM in heavily and lightly doped ZCO, respectively. 

In the next sections, it will be shown how the AIB model can account for other magnetic (i.e., magneto-optic) and non-magnetic ZCO properties. 

Before we leave the present section, we would like to comment on a possible effect of Co doping on sample magnetic anisotropy. In principle, magneto-crystalline anisotropy should not be affected by Co doping as long as there is no Co metal phase precipitation. In our samples (see Figure 3), both the coercive field Hc and the irreversibility field H* (i.e., the field at which the hysteresis cycle opens up) were found to increase with Co content: Hc = 80 (630) Oe for x = 0.04 (0.06), H* = 2.1 (3.6) kOe for x = 0.04 (0.06), indicating that magnetic anisotropy may be somewhat affected by the doping. The x = 0.06 sample had a higher fraction of metallic Co with respect to the other samples and different sizes of structurally coherent domains (20 nm vs. 12 nm for x = 0.04 [73]). Thus, microstructural characteristics can be invoked to explain this observed increase in magnetic anisotropy. 

### 3.2. Effects of Hydrogenation Treatments on the ZCO Magnetic Properties

#### 3.2.1. Increase in ZCO Ferromagnetism

The effects of the introduction of hydrogen atoms in semiconductor materials have been investigated extensively since pioneering studies showed the remarkable benefits that such a procedure has on a material’s transport and optical properties [81]. Generally, hydrogen atoms diffuse in the semiconductor lattice and compensate or passivate most deep and shallow defects purposely or accidentally present in the material. In group IV and III-V semiconductors, hydrogen often behaves as an amphoteric impurity, that is, like a donor in the presence of acceptors, and vice versa. In ZnO, instead, experimental [80,82] and theoretical [83] studies have shown that hydrogen acts exclusively as a shallow donor impurity. Hydrogen, as an unintentional impurity, may therefore be responsible for the *n*-type background conductivity of ZnO films [81]. However, it has been also suggested that increases in the conductivity and mobility observed in post-growth hydrogenated ZnO can have two different origins: improvements in mobility can be attributed to the passivation of the grain boundaries trap states present in polycrystalline films and induced by H atoms, while the increase in carrier concentration could be dominated by the formation of Zn interstitials also favored by the hydrogenation treatment [84,85].

Possible relationships between ferromagnetism and conductivity in H-irradiated ZCO were investigated in a combined experimental and theoretical study of hydrogenated polycrystalline ZCO thin films [25]. Magnetization versus magnetic field and carrier concentration measurements were performed at room temperature on four ZCO films (C1-C4) with a nominal Co content of 5%, irradiated with different H doses, with increasing doses from C1-H to C4-H samples (see Table 2). The magnetic measurements were also performed after a thermal annealing of the H-irradiated samples.

The main results of such measurements, i.e., the values of the *n* carrier concentration *N* and saturation magnetization *M_s_* measured for the as grown, H-treated, and (then) annealed samples, are reported in Table 2. Due to the shallow donor behavior of H in ZnO, in HZCO it was expected an increase of the *N* carrier concentration. Moreover, the above-mentioned positive correlations between the increase in *n* carrier concentrations and the FM increase induced by the addition of shallow donor dopants to ZCO [19,20,33,34,80] suggested an FM increase also in hydrogenated ZCO. The latter effect is fully confirmed by the results of Table 2. The *M_s_* value is indeed directly related to the H dose, it increases from 0.37 µ_B_/Co for C1−H up to 1.50 µ_B_/Co for the C4−H film. Moreover, an annealing at 500 °C caused a noticeable decrease in the values of *M_s_*, which returned to the value it had before the H treatment (Table 2). This result clearly shows that the significant *M_s_* increase observed upon hydrogenation is directly related to the presence of H or H-induced defects, which are removed by the annealing treatment. Instead, the values of the carrier concentration *N* showed quite puzzling behavior. First, they were not directly correlated with the H doses. Moreover, possible relationships between the values of the carrier concentration and saturation magnetization for the different H doses appeared somewhat complicated. For instance, the C1−H sample treated with the lowest H dose had the highest *N* value in correspondence with the lowest *M_s_* value. 

Interestingly, the AIB model introduced above fully accounts for the peculiar *N* trends and *N* vs. *M_s_* relationships shown by the results in Table 2, and clarifies how hydrogen can induce an increase in ZCO ferromagnetism. More specifically, first, it has to be taken into account that hydrogenation at high temperatures *increases the V_O_ concentration*, as shown by the XANES results [25]. In turn, an increase in the V_O_ concentration can induce an increase in the concentration of the Co-V_O_ complex. Then, the results in Table 2 can be considered in view of the acceptor character of the Co−V_O_ impurity band proposed in the AIB model. In the presence of shallow donors, such as Zn_int_s or H itself, this band can be populated with free CB electrons at the cost of the ZCO bulk electrical conductivity, as also reported in [24]. This implies that the carrier concentrations reported in Table 2 are a fraction of the carriers provided by the H shallow donors, that is, the fraction of carriers remaining in the CB after the filling of the Co−V_O_ impurity band. Only such free carriers contribute to the *N* values. These considerations explain the trends shown by the carrier concentrations and *M_s_* values of the hydrogenated samples reported in Table 2. In fact, for instance, in the C1−H sample, the lowest H dose induces the smallest concentration of Co−V_O_ complexes, thus resulting in a small saturation magnetization value, a small number of electrons filling the impurity band, and a large number of free electrons in the CB, i.e., high *N* and small *M_s_* values. By increasing the H dose, the concentration of Co−V_O_ complexes and the saturation magnetization value increase at the cost of the number of free electrons in CB. Finally, regarding the H effects on the ZCO magnetic properties, by assuming the AIB model, the increase in ferromagnetism in hydrogenated samples can be immediately explained by the two effects induced by the H introduction: the increases in V_O_ concentration and free electron density in CB.

#### 3.2.2. Improvements to ZCO Magneto-Optical Properties

In the context of DMSs, magnetic circular dichroism (MCD) [86] has a special role in understanding the strength and the nature of ferromagnetism as arising from spin-polarized carriers magnetically coupled to magnetic ions [86,87,88,89,90,91,92,93]. MCD stems from the magnetic field-induced Zeeman interactions of electronic structures [86], and it is a property shared by all materials in the presence a magnetic field applied along the direction of light propagation. The MCD technique, the differential absorption of left-handed circularly polarized (LCP) light and right-handed circular polarized (RCP) light, dates back to 1845 when M. Faraday discovered so-called magnetic optical rotation [94], which is due to the field-dependent difference in the refractive indices of LCP and RCP-transmitted light.

However, the presence of a finite optical absorption modifies the polarization of the transmitted light, giving rise to a rotation from linear (i.e., the superposition of LCP and RCP) components to elliptical. Therefore, the magneto-optical (MO) effect can be generally described by as a complex rotation whose real and imaginary parts are represented by Faraday rotation (rotation) and ellipticity (absorption), respectively. Such quantities provide information about the allowed optical transitions among the electronic states at energy E, split by the magnetic field according to the Zeeman effect. The MO effect was previously observed in Zn_1−x_Co_x_O films mainly at low temperatures [92,95]. Reports also exist of MCD at room temperature with hysteretic behavior at the band edge [96], and measurements of MCD after exposure to Zn metal vapor in a wide sub-bandgap range [97]. 

**Figure 7:**
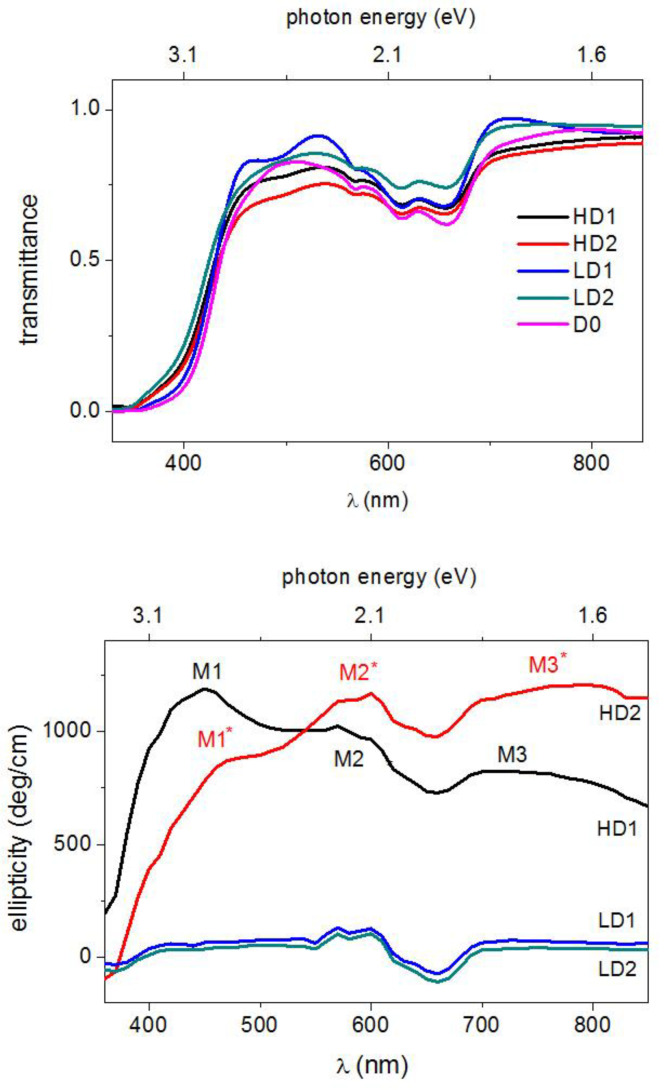
Transmittance (upper panel) and ellipticity spectra (lower panel) of the Zn_1−x_Co_x_O samples. Reproduced with permission from [74]. Copyright 2019 Royal Society of Chemistry.

As far as we know, the Faraday effect was not observed in Zn_1−x_Co_x_O, although results exist in the case of Ti-doped ZnO for photon energies larger than 3 eV [63]. The role of hydrogen on the optical and magneto-optical properties of ferromagnetic Co-doped ZnO films was addressed in [74] by measuring the room-temperature MCD spectra of Co-doped ZnO films irradiated by hydrogen ions. The HD1 and HD2 films were irradiated with a H^+^ dose of 4.5 × 10^18^ ions per cm^2^ (HD), and LD1 and LD2 were irradiated with 1.5 × 10^18^ ions per cm^2^ (LD). D0 was unirradiated. Some results reported in [74] are given in Figure 7, showing the room temperature transmittance curves and the MCD spectra (at the applied field of 1.3 T, calibrated in degrees (ellipticity) and normalized to the thickness) of the Zn_1-x_Co_x_O films. MO measurements were focused on the H-treated films. Both transmittance and ellipticity spectra exhibit a rapid increase near the band edge and a broad positive zone in the visible range up to 850 nm in the near infra-red (NIR) region, with some features near 600 nm. Absorption dips at about 566, 613 and 660 nm, originating from the electronic transitions of tetrahedrally coordinated Co^2+^ ions [6,95,98,99], are present in all transmission spectra.

**Figure 8:**
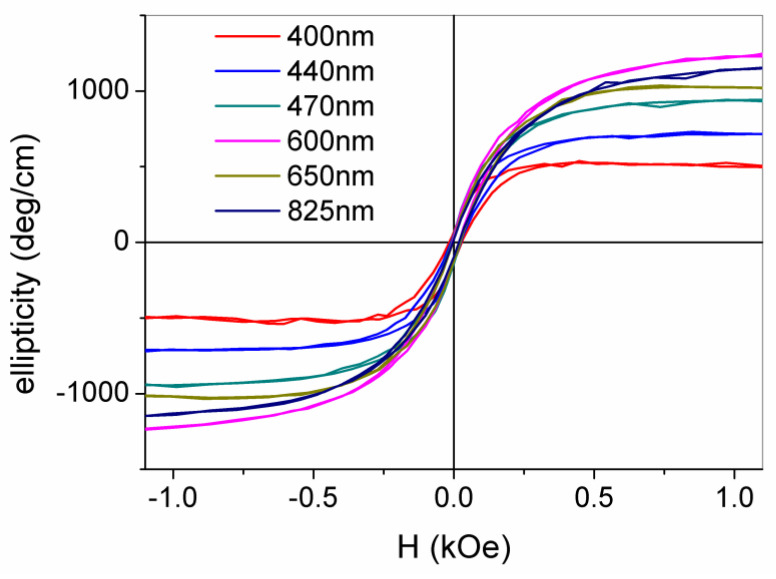
Room temperature magneto-optic hysteresis cycles of the HD2 films at different sub-bandgap wavelengths, starting from λ= 400 nm. Reproduced with permission from [74]. Copyright 2019 Royal Society of Chemistry.

This gives a clear indication that the added Co^2+^ ions have substituted Zn^2+^ cations without distorting the wurtzite structure in agreement with the results of a structural characterization. It should be noted that, unlike the transmittance curves which were quite independent of the H doping, ellipticity sharply increases with increasing hydrogen content, in line with the magnetization measurements. In fact, starting from values of about 100 deg/cm for LD1 and LD2 samples, the ellipticity exceeds 1000 deg/cm for the HD1 and HD2 samples in the range of 400–600 nm. This large positive value directly indicates that the magnetic interaction leads to a large splitting of the electronic states involved in the optical transitions and, hence, a large difference in the absorption of LCP and RCP light is expected. For the HD2 sample, the magnetic field dependence of the ellipticity (measured at wavelengths varying from the band edge up to 850 nm, in near-IR region) shows a clear hysteretic–ferromagnetic behavior (Figure 8), ruling out any paramagnetic character of the ion-carrier interaction.

Values of ellipticity at saturation ranges from 200 up to 1200 deg/cm with coercive fields of about 200 Oe, in fair agreement with the measured magnetization values. In the sub-bandgap region (λ > 375 nm), the saturated ellipticity is observed to increase with λ up to 600 nm, where a value of 1200 deg/cm is found, keeping high values except for λ values corresponding to the optical absorption of the Co^2+^ ions. 

The hysteretic behavior of the ellipticity vs. the magnetic field curves confirmed the intrinsic nature of the ferromagnetic coupling originating from a spin-dependent band structure, with interaction between the carriers and the localized spin of the magnetic ions. In the Zn_1-x_Co_x_O case, Co ions could not sustain magnetic interactions beyond their first neighbors [32], whereas, as suggested by the AIB model, the Co-V_O_ complexes can promote the long-range ferromagnetic order if additional *n* doping is present [20,24,32]. More specifically, the strength of the magnetic interaction increases by filling the acceptor-like band due to Co-V_O_ complexes with carriers arising from donor defects [24,32]. Hence, the results of the higher MCD values for the films treated with high H doses agreed well with the AIB model, as H irradiation induces additional carriers and favors the formation of V_O_ defects [25]. 

The ellipticity rapidly increased near the band edge and kept high values in the sub-bandgap region; such behavior is associated with photoionization transitions involving electronic states induced by Co-defect complexes in the ZnO energy gap [21]. The HD1 and HD2 ellipticity spectra of Figure 7 (bottom panel) show different shapes characterized by different relative maxima placed approximately at 430 nm (2.88 eV), 600 nm (2.10 eV), and in the range of 700–800 nm (1.77–1.55 eV).

**Figure 9:**
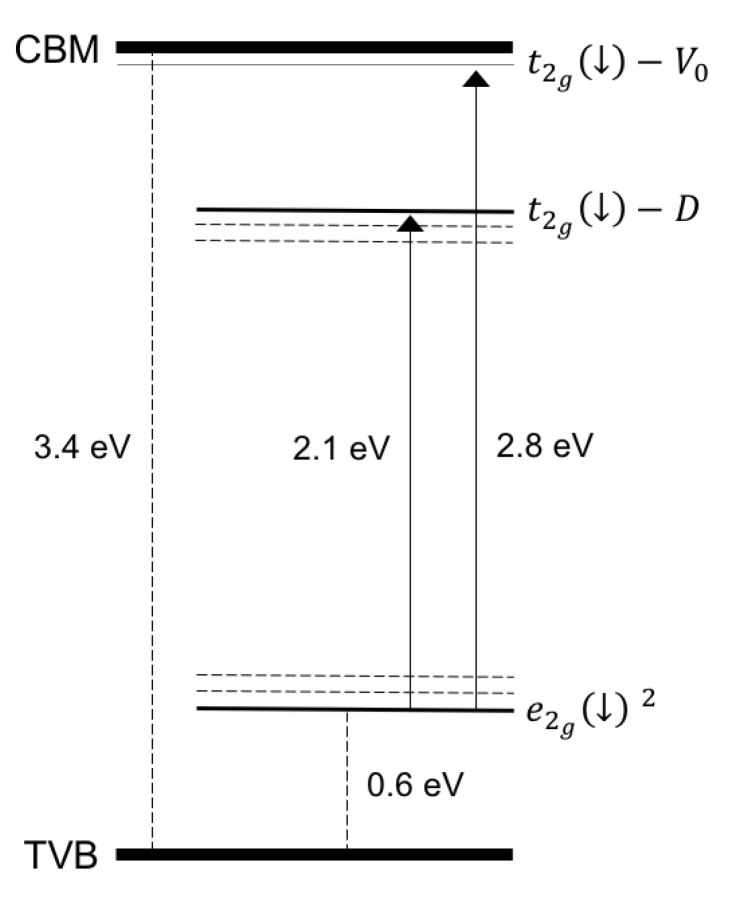
Schematic diagram showing electronic states induced by Co–V_O_ and Co–D complexes (see the text) in the ZnO energy gap. TVB and CBM indicate the top of the valence band and the conduction band minimum, respectively. Reproduced with permission from [74]. Copyright 2019 Royal Society of Chemistry.

Even the fine structures of such spectra were explained in a phenomenological way on the basis of the above AIB model, which provided indications about the levels induced by the Co-V_O_ complexes in the ZnO electronic structure (see sketch in Figure 9). In agreement with the results discussed in Section 3.1 and schematized in Figure 2, Figure 9 shows the location of the *t*_2_^↓^ states induced by the Co-V_O_ complexes (*t*_2g_^↓^-V_O_ states in the figure), which were close to and partially mixed with the CBM when populated with electrons provided by shallow donor defects, as well as the filled *e*_2g_^↓^ states. In addition, the figure reports that *t*_2g_^↓^-D electronic states were deeper in energy than the *t*_2g_^↓^-V_O_ ones. The existence of these novel states was suggested by a KPFM (Kelvin Probe Force Microscopy) study [77] which showed a significant Fermi level lowering of about 0.4 eV in Zn_1−x_Co_x_O, indicating the presence of Co-induced deep levels in the ZnO energy gap (see also the following Section 3.3.2). Such levels were related to the formation of Co-defect (Co–D) complexes which enhance the Co under-coordination already realized in the Co–V_O_ complex, thus inducing a further lowering of the *t*_2g_^↓^-V_O_ state and giving rise to the *t*_2g_^↓^-D state of Figure 9.

Thus, the Co–V_O_ and Co–D complexes may induce two kinds of electronic states close to the CBM and in the energy gap, respectively. These states, under illumination with light of a suitable wavelength (approximately in the range of 3.0–1.5 eV), become empty or partially occupied, and therefore available for internal d–d optical transitions starting from the *e*_2g_^↓^-filled states common to Co and its complexes. Experimental and theoretical results located the *t*_2g_^↓^-V_O_ state very close to the CBM, whereas the KPFM results only indicated that the *t*_2g_^↓^-D states were approximately located below the Fermi energy. They were roughly located at 0.7 eV below the CBM, as shown in Figure 9. It was also considered that the Co–D complex can represent a family of complexes where the Co atom undergoes different degrees of under-coordination. This, and an expected structural, local disorder, can induce a small dispersion of these states, as well as of the corresponding *e*_2g_^↓^-filled states in a certain range of energy, schematized by dashed lines in the same figure. The HD1 and HD2 ellipticity spectra were explained in a phenomenological way on the basis of the electronic states sketched in Figure 9 and assuming different relative concentrations of the Co–V_O_ and Co-D complexes in the HD1 andHD2 samples. In detail, it was considered that the high-energy side of the HD1 ellipticity signals reaches a first maximum value M1 at about 430 nm, when the transmission of light becomes high (E < 3 eV) (the MCD measurement is performed in transmission). The photon energy corresponds to the energy required to bring *e*_2g_^↓^ electrons to the corresponding *t*_2g_^↓^- V_O_ level, about 2.8 eV higher in energy (430 nm). As soon as the energy becomes small enough for such transitions, the HD1 ellipticity signals decrease and then reach a second, relative maximum (M2) at about 580 nm, 2.1 eV, that may correspond to the transitions from *e*_2g_^↓^ to the *t*_2g_^↓^-D level. Then, a further signal decrease is followed by reaching the third relative maximum (M3) corresponding to a sort of plateau in the range of 700–800 nm, that is, 0.4–0.6 eV below the M2 peak. This range may still correspond to the *e*_2g_^↓^→ *t*_2g_^↓^-D transition once the small dispersion in the energy of the *e*_2g_^↓^ and *t*_2g_^↓^-D states (Figure 9) is taken into account. The different amplitudes of the M1, M2, and M3 peaks can be explained by a predominant presence of the Co–V_O_ complexes with respect to the Co-D ones in the HD1 sample. For HD2, the ellipticity shows three relative maxima (M1*, M2*, and M3*) basically at the same energies of the M1, M2, and M3 maxima. Such peaks correspond, therefore, to the same transitions explaining the HD1 spectrum, while the different MX* vs. MX amplitudes may be explained in terms of the different relative concentrations of the Co–V_O_ and Co-D complexes in the two samples, the latter complex being the predominant one in the HD2 sample. Such a successful interpretation of the ellipticity spectra further strengthens the reliability of the AIB model. 

**Figure 10:**
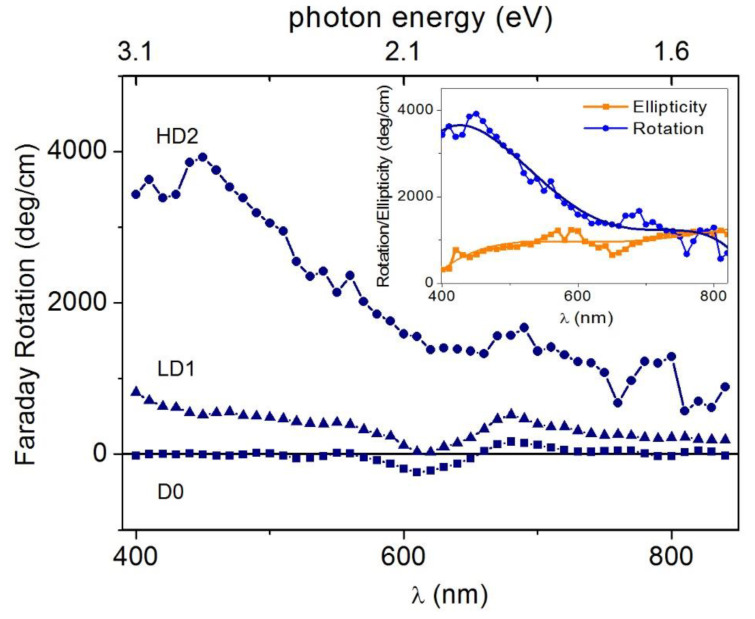
Room temperature Faraday rotation at 1.3 T for unirradiated D0, and the low (high) H-dose-treated sample LD1 (HD2) film. Inset: Faraday rotation and ellipticity for the HD2 sample; lines are a guide to the eye. Reproduced with permission from [74]. Copyright 2019 Royal Society of Chemistry.

The ellipticity also received contributions from the differential RCP-LCP light absorption at 566, 613, and 660 nm wavelengths associated with the optical Zeeman-split d*-d transitions. By the inspection of Figure 7 we identified that, for the LD1 and LD2 samples, the ellipticity took negative values at around 600 nm. This indicates that the optical d*-d transitions give the main contribution to the ellipticity as the H dose, and hence the carrier density in LD1 and LD2 are consistently lowered with respect to HD1 and HD2, thus reducing the photoionization. In the broad perspective of opto-spintronics devices [100], the complex Faraday rotation spectra at saturation (Figure 10) was also measured for unirradiated D0 and the low (high) H-dose-treated sample LD1 (HD2).

Indeed, the LD1 and LD2 samples exhibited a sizeable effect, reaching giant values for HD2, whereas the MO response to D0 was quite negligible. This gives a further proof that the electronic structure of ZCO and, hence, its MO response, can be effectively modified by H irradiation. Fixing the attention on the HD2 sample (inset of Figure 10), it can be noted that a pure rotatory regime occurred from the band edge up to 550 nm, where a rotation of the polarization plane up to 4000 deg/cm was observed at around 368 nm (corresponding to the ZnO exciton wavelength). For λ > 550 nm, the ellipticity reached the rotational value θs of 1000 deg/cm, with a linear to elliptical switching of the polarization of transmitted light when λ > 700 nm.

#### 3.2.3. Local MO Response

**Figure 11:**
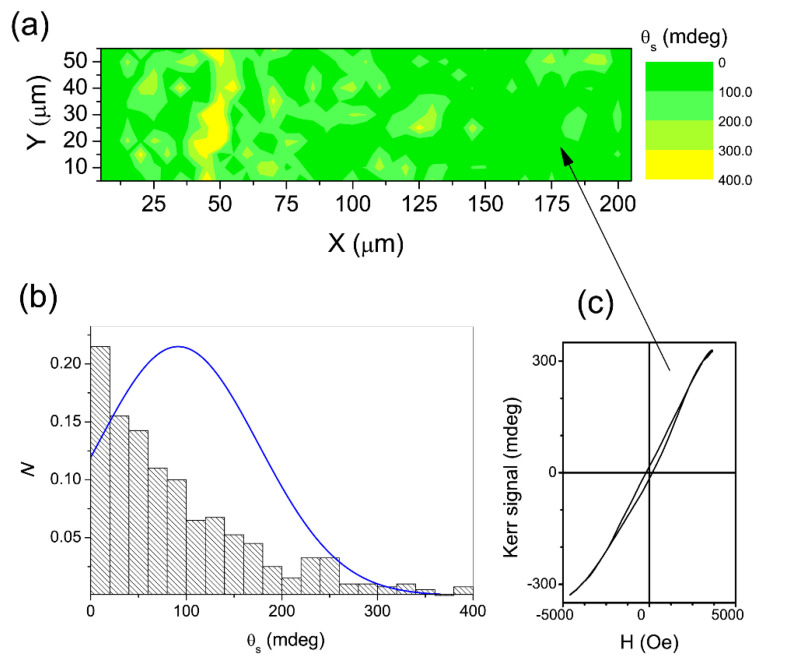
(**a**) Map of the Kerr rotation values at saturation. (**b**) Histogram representing the density of the sampled cells for a unit bin interval with a normal distribution fit (blue line) to the data. (**c**) Kerr hysteresis loop of the microzone (5 × 5 μm^2^) is indicated on the map by the arrow. Reproduced with permission from [101]. Copyright © 2022, EDP Sciences, Springer-Verlag GmbH Germany, part of Springer Nature Copyright 2019.

By measuring the Kerr rotation at the saturation (θ_s_) of the ZCO film, a map was constructed (Figure 11a, upper panel) showing that the local θ_s_ values were mostly below 100 mdeg, with a minor number of domains with θ_s_ larger than 200 mdeg and only a few spots with θ_s_ exceeding 300 mdeg. The local θ_s_ distribution (Figure 11b, lower panel) was fitted with a Gaussian function, with an average < θ_s_ > of about 90–100 mdeg and a standard deviation of ~40 mdeg.

**Figure 12:**
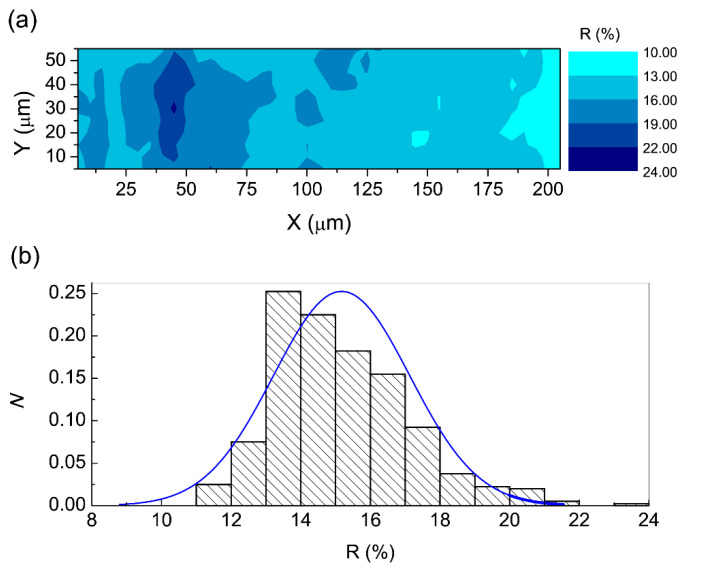
(**a**) Film reflectivity map. (**b**) Histogram of the density of sampled cells for a unit bin interval with a normal distribution fit (blue line) to data. Reproduced with permission from [101]. Copyright © 2022, EDP Sciences, Springer-Verlag GmbH Germany, part of Springer Nature Copyright 2019.

As a result of the high film transmittance, [74] it was assumed that the whole film contributed to the Kerr signal at 660 nm, allowing us to estimate a value of about 1900 deg/cm for the θ_s_ per unit length, which was consistent with the FR measurements [74]. The Kerr hysteresis loop of the microzone (5 × 5 μm^2^) (indicated on the map by an arrow; Figure 11c) was further proof of the ferromagnetism in the film. Moreover, by inspecting the film reflectivity (R) (Figure 12a), it transpires that the largest fraction of the area reflects less than 15–16%, with small, isolated areas of about 20%. The histogram of Figure 12b shows that the R values fit a normal distribution with an average value <R> of 15%, suggesting that randomly distributed grains should be responsible for reflection. When we compare the reflectivity and Kerr maps, it transpires that the high-R areas correspond to high-θ_s_ areas. This should be ascribed to a donor-like impurity band due to the concurrent presence of O vacancies to form complexes with Co and carriers from shallow donors [74].

As a final remark, it should be noted that the investigation of the MO response at both macroscopic and microscopic scales strongly supports the effectiveness of the hydrogen treatment in modifying the electronic structure of ZCO which, in turn, is at the heart of the observed room temperature ferromagnetism.

### 3.3. Other Effects of Co Doping and H Co Doping on the ZnO Properties

Although the present review is devoted to the magnetic and magneto-optical properties of ZCO and HZCO, in the next sections we briefly discuss other material properties which can be consistently accounted for by the AIB model.

#### 3.3.1. Transport Properties in ZCO and Hydrogenated ZCO

**Figure 13:**
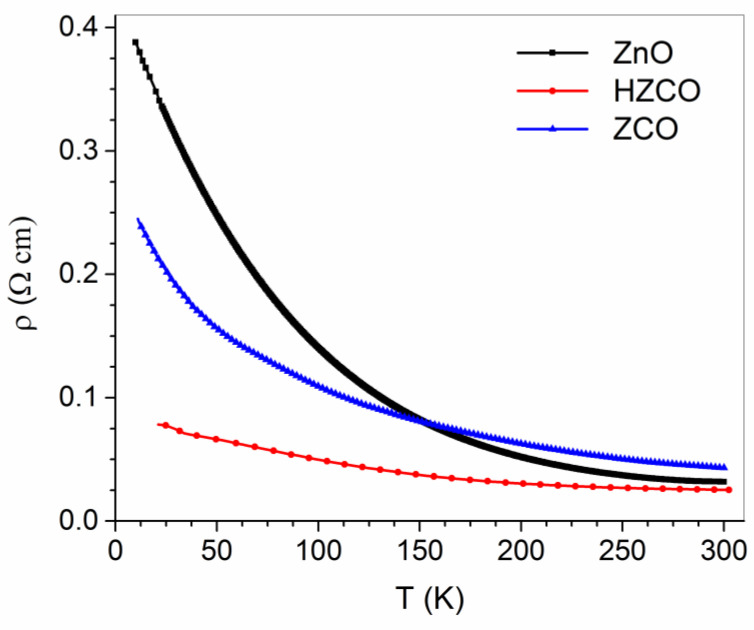
Resistivity (ρ) as a function of temperature for ZnO, ZCO, and HZCO films in the range 20–300 K. Reproduced with permission from [76]. Copyright 2021 Royal Society of Chemistry.

In a previous study [76], the temperature behavior (20–300 K range) of electrical resistivity (ρ) was investigated in polycrystalline ZnO, Co-doped ZnO (ZCO), and H-irradiated ZCO (HZCO) samples. The results showed stunning effects of Co doping and H irradiation on the ZnO transport properties. In detail, the Co dopant increased the ZnO resistivity at high T (HT), whereas it had an opposite effect at low T (LT). Atomic H balanced the Co effects by neutralizing the ρ increase at HT and strengthening its decrease at LT (Figure 13). The same study identified two different thermally activated processes as responsible for the charge transport in the three materials at HT and LT, respectively. Remarkably, the occurrence of such processes was fully accounted for by the AIB model and the effects produced by the introduction of atomic H on the ZCO properties discussed in Section 3.2.1.

#### 3.3.2. Effects of Co Doping on the ZnO Surface Reactivity

In a previous study [77], to gain an insight into the changes in electronic structure and surface reactivity induced by the Zn substitution with Co, scanning probe microscopy studies of ZnO and Co-substituted ZnO under dark/UV conditions were performed both in air and an ultra-high vacuum. Two major results were achieved: first, the Co substitution of Zn atoms significantly downward shifted the Fermi level by about 400 meV, which was close to the conduction band in the as-grown *n*-type ZnO. Second, the absorption of negative oxygen species (NOS) at the ZnO surface was strongly reduced by Co substitution. 

Remarkably, these two effects of Co doping were clearly explained by the AIB model. In detail, an unoccupied Co-D electronic band was assumed to exist in the ZnO energy gap, where D stands for a single or double oxygen vacancy, that is, a defect that leads to a significant under-coordination of the Co atom. This band may host electrons released by unintentional shallow donors, hence reducing their density in the CB. This accounts for both the suppression of NOS, as adsorbed molecules are charged and stabilized by the availability of free electrons in the CB, and the downward shift of the Fermi level. The existence of the Co-D electronic band has subsequently received further support due to its full consistency with the results of the ZCO magneto-optical investigation discussed in Section 3.2.2. 

## 4. Conclusions

In the present paper, we discussed the results of previous studies on the magnetic and magneto-optical properties of Co-doped and hydrogen-irradiated Co-doped polycrystalline ZnO thin films. We also briefly recalled the results of investigations into the transport and surface properties of the same materials. Drawing our conclusions, we want to focus on the interpretation of all of these experimental results. Let us start by acknowledging that, considering the occurrence of ferromagnetism in lightly doped ZCO and HZCO, an explanation of the experimental findings reported above has to take into account the following facts: (i) RT-FM in ZCO is related to the presence of V_O_s, (ii) Co doping causes a reduction in the *n* carrier concentration as though a substitutional Co in ZnO could induce the formation of some kind of acceptor defect (let us note that the latter effect should be a very strange consequence of Co doping, because a Zn atom is substituted with an iso-electronic atom which is also quite close in size), and (iii) RT-FM is enhanced by the presence of shallow donors. In regard to these facts, the acceptor–impurity–band model, suggested by the theoretical results, does not require the formation of any kind of acceptor defects. In the model, an acceptor behavior originates simply by the combination of a Co atom with a native or induced V_O_. In fact, the formation of a Co-V_O_ complex reduces the Co coordination, thus weakening the effect of the surrounding crystal field on the Co 3*d* orbitals whose energy levels are initially located in the conduction band. This induces a descent of such levels which, once occupied by electrons provided by shallow donors, reach a location close and slightly below the conduction band minimum. Thus, the AIB model fully agrees with the three instances of factual data above. It also fully accounts for all of the experimental results reported above, thus permitting their interpretation in a unifying theoretical framework, allowing the clear explanation of RT-FM in lightly doped ZCO, which is the major indication given by the present review.

## Figures and Tables

**Table 1:** Experimental values of some magnetic, structural and electrical parameters for the C0, C2, C3, and C4 films. Values of the magnetization M_s_ and estimates of the Co metal and Co–V_O_ complex concentrations for the C2 and C4 films are taken from [73]. Reproduced with permission from [24]. Copyright 2015 American Chemical Society.

	C_0_	C_2_	C_3_	C_4_
**M_s_ (emu/cm^3^)** (mean error ± 0.45)	0.0	0.80	2.22	5.08
**M_s_ (μ_B_/cm^3^)** (mean error ± 0.45 × 10^20^)	0.0	0.86 × 10^20^	2.40 × 10^20^	5.48 × 10^20^
**Co-V_O_** (% of Zn ions) (mean error ± 0.2)	*n.d.*	0.0	0.6	1.2
**Co-V_O_ (cm^−3^)** mean error ± 0.9 × 10^20^	*n.d.*	0.0	2.0 × 10^20^	4.0 × 10^20^
**Co metal** (% of Co ions) (mean error ± 2)	*n.d.*	0.1	2.1	3.7

**Table 2:** Conductive and magnetic parameters for the samples C1, C2, C3, and C4 irradiated with four different H doses. C stands for 10^18^ ions/cm^2^. Reproduced with permission from [25]. Copyright 2016 American Chemical Society.

Samples	*N* (cm^−3^) *as grown*	M_s_ (μ_B_/Co) *as grown*	*N* (cm^−3^) *H-treated*	M_s_ (μ_B_/Co) *H-treated*	*N* (cm^−3^) Annealed @250 °C	M_s_ (μ_B_/Co) Annealed @250 °C	*N* (cm^−3^) Annealed @500 °C	M_s_ (μ_B_/Co) Annealed @500 °C
C1 d_H_ = 1.5C	2.8 × 10^18^	0.22	1.2 × 10^19^	0.37	6.2 × 10^18^	0.41	<10^16^	0.26
C2 d_H_ = 3C	2.0 × 10^18^	0.23	3.4 × 10^18^	0.85	---	---	---	---
C3 d_H_ = 4.5C	2.7 × 10^18^	0.23	5.6 × 10^18^	1.10	9.2 × 10^18^	0.78	<10^16^	0.24
C4 d_H_ = 6C	2.0 × 10^18^	0.23	5.2 × 10^18^	1.50	---	---	---	---

## Data Availability

Data supporting reported results are available upon requesting to A.D.T.
